# Luteinizing Hormone/Human Chorionic Gonadotropin Receptor N312S Single-Nucleotide Polymorphism and Its Impact on Clinical and Reproductive Outcomes in Assisted Reproductive Technology: A Prospective Cohort Study

**DOI:** 10.7759/cureus.47217

**Published:** 2023-10-17

**Authors:** Amulya Nagulapalli, Radha Vembu, Monna Pandurangi, Sanjeeva R Nellepalli

**Affiliations:** 1 Department of Reproductive Medicine and Surgery, Sri Ramachandra Institute of Higher Education and Research, Chennai, IND

**Keywords:** assisted reproductive technology, clinical outcomes, reproductive outcomes, genotypic distrubution, lhcgr n312s snp

## Abstract

Objective

The aim of this study was to determine the genotypic distribution of luteinizing hormone/human chorionic gonadotropin receptor (LHCGR) N312S single-nucleotide polymorphism (SNP) and to investigate its impact on clinical and reproductive outcomes in infertile Indian women undergoing assisted reproductive technology (ART).

Study design and settings

This was a prospective cohort study conducted at a tertiary care university hospital.

Subjects and methods

Infertile women aged between 21 and 40 years undergoing ART with an antagonist protocol were enrolled in this study. A 2-ml sample of peripheral venous blood was collected from each woman and genotyped for the LHCGR N312S SNP. Participants were divided into three groups based on their SNP: NN, NS, and SS. All subjects underwent controlled ovarian hyperstimulation (COH) through a gonadotropin-releasing hormone (GnRH) antagonist protocol and intracytoplasmic sperm injection (ICSI). Of the 140 women recruited based on selection criteria, 128 underwent embryo transfer. We compared the genotypic distribution of the LHCGR N312S SNP, baseline characteristics, clinical outcomes, and reproductive outcomes in ART among the three groups. Data were analyzed using IBM SPSS Statistics for Windows, Version 29 (Released 2022; IBM Corp., Armonk, New York, United States). The chi-square test and Fisher-Irwin test were employed to evaluate significant differences among the qualitative categorical variables. A p-value of less than 0.05 was considered statistically significant.

Results

Among the test subjects, 19.3% were homozygous for the LHCGR N312 SNP (NN group), 38.6% were heterozygous (NS group), and 42.1% were homozygous for the LHCGR S312 SNP (SS group). Baseline characteristics were similar among the three groups. In terms of ovarian reserve tests, significantly lower anti-Müllerian hormone (AMH) levels were observed in the SS group compared to the NS and NN groups (2.8 ± 2.1 vs. 3.2 ± 2.5 vs. 4.3 ± 3.3; p=0.03). No significant differences were observed in COH outcomes such as duration of stimulation, total gonadotropin requirement, oocyte yield, or the number of good-quality embryos among the three groups. The cumulative pregnancy rate (82.9% vs. 50.0% vs. 38.2%, p=0.0005), cumulative clinical pregnancy rate (78.8% vs. 44.7% vs. 34.5%, p = 0.0005), and cumulative live birth rate (50.0% vs. 20.2% vs. 20.0%, p=0.005) were significantly higher in the NN group than in the NS and SS groups.

Conclusion

The study’s findings suggest that LHCGR N312 may help predict reproductive outcomes in ART, which may aid in providing better counseling to infertile couples. We need more studies on individualized/personalized COH using pharmacogenomics for follicle-stimulating hormone (FSH) and luteinizing hormone (LH) supplementation based on combined FSH and LH receptor SNP and to assess their effects on ART outcomes.

## Introduction

Infertility is defined as "failure to establish a clinical pregnancy after 12 months of regular unprotected sexual intercourse" [1]. It affects approximately 13-15% of couples worldwide [2]. The prevalence of infertility has increased over the past two decades, mainly due to changes in environmental factors, social factors such as delayed childbearing, lifestyle changes, socioeconomic factors, and genetic factors. Assisted reproductive technology (ART) plays a crucial role in addressing the reproductive challenges faced by couples unable to conceive.

In ART, the treatment goal is to safely retrieve an adequate number of mature oocytes to achieve a live birth [3]. Controlled ovarian hyperstimulation (COH), which involves using exogenous gonadotropins like follicle-stimulating hormone (FSH) and luteinizing hormone (LH) to obtain mature oocytes, is an important step in ART. The dose of gonadotropins in COH is usually individualized based on various factors such as age, body mass index (BMI), prior response to ovarian stimulation, and ovarian reserve tests like antral follicle count (AFC) and anti-Müllerian hormone (AMH) [4,5]. There is variation in individual responses to COH, and 24% may have a suboptimal or unexpectedly poor response that basal characteristics and ovarian reserve tests alone cannot predict; genetics may play a role here [6].

Nucleotide changes in glycoprotein hormones and their receptors can modify the response to COH. Genetic polymorphism is defined as the presence of two or more alleles at one locus with considerable frequency and a minimum frequency of 1% [7].

Common genetic variants, such as single-nucleotide polymorphisms (SNPs) of gonadotropins and their receptors, can affect ovarian response to COH and ART outcomes. Pharmacogenomics is the study of how genes influence responses to medication [8]. Tailoring gonadotropin therapy based on pharmacogenomics could help achieve optimal outcomes in ART.

Most SNPs contain two alleles. An allele is referred to as a "major" allele if the SNP is more prevalent in the general population, and as a "minor" allele if the SNP is less common. Because humans are diploid, an individual can have different genotypes, such as being homozygous for the major or minor allele or heterozygous for major and minor alleles [9].

LH mediates its effects through luteinizing hormone/human chorionic gonadotropin receptors (LHCGR) located on granulosa cells, theca cells, and the corpus luteum [10]. LH and human chorionic gonadotropin (hCG) exert their actions via the same receptor. LHCGR, like the follicle-stimulating hormone receptor (FSHR), is a G-protein-coupled receptor with 7-transmembrane helices and is required for follicle maturation, maintenance of theca cells, and ovulation. In theca cells, LH via LHCGR aids in producing androstenedione, which aromatase subsequently converts to estradiol in granulosa cells. In granulosa cells, it causes ovulation, luteinization, and the formation of the corpus luteum.

The gene for LHCGR is situated on chromosome 2 near the FSHR gene and comprises 11 exons [11]. Among the known SNPs in the LHCGR gene, N312S (rs2293275) SNP in exon 10 has been extensively studied [11]. The A (Adenine) is replaced by G (Guanine) at position 312 of exon 10 in the LHCGR gene, replacing asparagine (N) with serine (S). If there are two A alleles at the 312 position, they are grouped as homozygous for N312 (NN). Substitution of one A allele with G leads to heterozygous for the N312S genotype (NS). If both A alleles are replaced by G, they are homozygous for S (SS). Because this polymorphism is found close to the glycosylation site in the extracellular domain of LHCGR, changes in sequence might impact receptor sensitivity. Several researchers have discovered a relationship between N312S and polycystic ovarian syndrome (PCOS), with the N variant making LHCGR more sensitive [12-14].

Both LH and FSH are required for adequate oocyte maturation; hence, variants of LHCGR play a role in ART. The production of homo- and heterodimers of the FSHR and LHCGR receptors explains how activation by one hormone can mediate the response via the receptor of the other hormone. As a result, the LHCGR genotype may alter the response to FSH stimulation [11].

There are few data on the LHCGR gene's N312S SNP and its effect on ART outcomes, especially in the Indian infertile population, and the results are contradictory.

The aim of our study was to investigate the genotypic distribution of the LHCGR N312S SNP and how it affects clinical and reproductive outcomes in women undergoing ART in the infertile Indian population.

## Materials and methods

Study population

This was a prospective cohort study conducted in the Department of Reproductive Medicine and Surgery of a tertiary care university hospital from December 2021 to April 2023. Inclusion criteria were females aged 21-40 years undergoing ART with gonadotropin-releasing hormone (GnRH) antagonist protocol. The study excluded patients with hypogonadotropic hypogonadism and patients with a history of chemotherapy or radiotherapy.

Sample size

The sample size estimate was 96. The study was approved by the Institutional Ethical Committee (CSP-MED/21/NOV/72/139). Patients enrolled in the study provided written informed consent.

Methodology

Infertile women who met the selection criteria were recruited for the study. Prior to stimulation, 2 ml venous blood samples were obtained, and DNA extraction with subsequent LHCGR genotyping for LHCGR N312S SNP was performed (Table 1).

**Table 1:** Steps of genotyping for LHCGR N312S single-nucleotide polymorphism (SNP)

Steps of Genotyping	
Source of sample	2 ml of peripheral venous blood collected in ethylenediaminetetraacetic acid (EDTA) vacutainer
DNA extraction	Isolated from leukocytes by modified salting out method
Polymerase chain reaction (PCR) amplification	LHCGR gene on Exon 10 was amplified by PCR with 10 mM primers and 2x master mix consisting of Taq DNA forward primer: 5’GACAATGGTGCAGAACGAGATG3’, Reverse primer: 5’GCAACAGCTCCGTAACCAAGAC3'. The amplification was performed with an initial denaturation at 95^o^C for 10 minutes, followed by 30 cycles of denaturation at 95^o^C, annealing at 60^o^C for 20 seconds and 72^o^C for 20 seconds followed by a final extension at 72^o^C for 5 minutes
Gel electrophoresis	On 2% agar gel
Product clean-up	Using exonuclease I and shrimp alkaline phosphatase (ExoSAP-IT)
Sequencing	Sequenced using a big dye cycle sequencing kit on a 3500 Genetic Analyzer
Analysis	Done using SeqScape: On exon-10 of chromosome 2, SNP at position 312, N312S (rs2293275, allele change: AAT to AGT, reference sequence: NM_000233.3: c.935A > G) replacing asparagine (N) with serine (S) was determined

Ovarian stimulation protocol

A GnRH antagonist protocol was used for COH. Prior to ovarian stimulation, medical care professionals were unaware of the polymorphism genotyping report. On day 2 of the cycle, COH was initiated with recombinant FSH (rFSH, Gonal F®, Merck Serono S.A., Switzerland) and/or human menopausal gonadotropin (HMG, Gynogen® HP, Sanzyme Private Limited, Hyderabad, India) and/or urinary FSH (uFSH, Folliculin™, Bharath Serum and Vaccines Limited, India), and the dose was tailored for each patient based on age, BMI, AFC, AMH levels, and/or prior response to ovarian stimulation. A transvaginal ultrasound scan (TVS, 7MHZ) was used to assess follicular development on the 6th day of COH, and the gonadotropin dose was adjusted to achieve optimal response. GnRH antagonists such as cetrorelix 0.25 mg/day subcutaneous (Asporelix™ 0.25mg, Bharath Serum and Vaccines Limited, India) were commenced when the leading follicle was ≥ 14 mm and continued until the day of triggering. When two or more follicles were ≥18 mm, a dual trigger was given in all patients except those with hyperresponses when a GnRH agonist trigger was given for ovulation. Oocyte retrieval was performed 34-36 hours after triggering under TVS guidance. In all patients, intracytoplasmic sperm injection (ICSI) was performed. Istanbul consensus was used for embryo grading until the cleavage stage, and Gardner’s criteria were used for blastocyst grading. According to institutional protocol, patients had either fresh or frozen embryo transfer (FET).

Fresh embryo transfer

Estradiol valerate twice daily (Progynova® 2 mg, Bayer Zydus Pharma AG, Germany) and inj. micronised progesterone 100 mg (Susten® 100, Sun Pharma Laboratories Limited, Mumbai, India) intramuscularly/day was started on the day of retrieval for three days in cleavage stage embryo and for five days in blastocyst transfer. Embryo transfer was performed under transabdominal ultrasound guidance using a Cook catheter (Cook Medical, Bloomington, USA). Micronized vaginal progesterone 200 mg (Susten® 200, Sun Pharma Laboratories Limited, Mumbai, India) thrice daily, and dydrogesterone 10 mg (Abott India Limited, Puducherry, India) thrice daily orally were administered as luteal support.

Frozen embryo transfer

Based on patient characteristics, direct hormone replacement therapy (HRT) or suppressed cycle HRT was performed in frozen embryo transfer cycles. In suppressed cycle HRT, pituitary suppression was achieved with a combined oral contraceptive pill (OCP-ethinyl estradiol 0.03 mg + levonorgestrel 0.15 mg) given from the second or third day of the next menstrual cycle after oocyte retrieval for 21 days. Injection leuprolide acetate depot 3.75 mg was given subcutaneously on day 14 of OCP. After withdrawal bleeding, on day 5 of the cycle, HRT was started with 4 mg of estradiol valerate per day for five days, followed by 6 mg for six days in suppressed cycle HRT, and in direct HRT cycles from day 2/3, estradiol valerate 6 mg per day was started. Endometrial thickness (ET) and pattern were assessed by TVS. Estradiol valerate dose was adjusted until ET ≥ 8 mm, and luteal support was given as in a fresh embryo transfer. Serum beta HCG was done two weeks after embryo transfer and if it was ≥ 5 mIU/ml, TVS was performed four weeks post-embryo transfer to assess for viability, and luteal support was continued up to 10 weeks. All women were followed up for a maximum of two embryo transfer cycles from conception until delivery.

Definition of reproductive outcomes

Serum beta HCG ≥ 5 mIU/ml is defined as pregnancy. Clinical pregnancy is defined as the detection of an intrauterine gestational sac with cardiac activity on TVS at seven weeks’ gestation. The live birth rate is defined as the birth of at least one newborn after 24 weeks. Miscarriage was defined as pregnancy loss during the first trimester. The ongoing pregnancy rate is defined as pregnancy continuing beyond 20 weeks. The cumulative pregnancy rate (number of patients with beta-HCG positive/total number of embryo transfers), cumulative clinical pregnancy rate (number of patients with detection of intrauterine gestational sac with cardiac activity on TVS/total number of embryo transfers), cumulative miscarriage rate (number of miscarriages/total number of pregnancies in two embryo transfers), and cumulative live birth rate (number of deliveries that resulted in live born neonate after 24 weeks expressed per 100 embryo transfers up to two FET cycles) were recorded.

Statistical analysis

IBM SPSS Statistics for Windows, Version 29 (Released 2022; IBM Corp., Armonk, New York) was used to analyze the collected data. The categorical variables were represented with the help of frequency analysis as well as percentage analysis. Mean ± standard deviation was used to report continuous variables. An independent sample t-test was used to determine the significant difference between bivariate samples in the independent group. The chi-square test and Fisher's Irwin Test were used to evaluate the significant difference among the qualitative categorical variables. A p-value of less than 0.05 was considered statistically significant.

## Results

**Figure 1:**
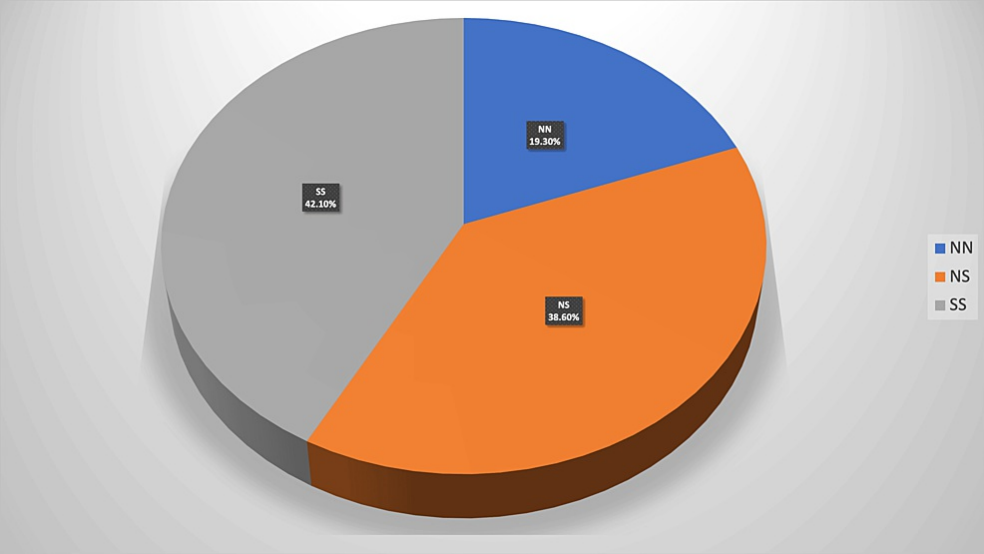
LHCGR N312S single-nucleotide polymorphism (SNP) genotypic distribution

The study included 140 infertile women undergoing ART who satisfied the inclusion and exclusion criteria, of which 128 women had undergone embryo transfer. The patients were divided into three groups based on nucleotide sequence at 312: NN, NS, and SS groups. Among the 140 women,19.3% (NN, n=27) were homozygous for N312, 42.1% (SS, n=54) were homozygous for S312, and 38.6% (NS, n=59) were heterozygous for N312S (Figure 1).

The baseline characteristics were comparable among the three groups. There were significant differences in AMH levels among the three groups, with lower AMH levels in the SS group when compared to the NN and NS groups (Table 2).

**Table 2:** Baseline characteristics ^a^Percentage ^b^Mean±SD ^c^p<0.05 ART, assisted reproductive technology; AFC, antral follicle count; FSH, follicle-stimulating hormone; AMH, anti-Müllerian hormone

Baseline Characteristics	Whole Cohort (n=140)	NN (n=27)	NS (n=54)	SS (n=59)	P-value
Age (years)^b^	31.6±4.0	31.4±4.3	31.1±3.6	32.2±4.3	0.39
BMI (kg/m^2^)^b^	26.8±4.6	25.7±5.1	26.8±4.8	27.2±4.2	0.36
Duration of infertility (years)^b^	6.3±3.5	5.7±2.9	6.1±3.2	6.8±4.0	0.38
Indications for ART					
Female factor^a^	36.4%	33.3%	35.2%	39.0%	
Male factor^a^	22.2%	37.1%	18.5%	18.6%	0.25
Combined factor^a^	30.7%	22.2%	33.3%	32.2%	
Unexplained factor^a^	10.7%	7.4%	13.0%	10.2%	
Type of infertility					
Primary^a^	73.6%	81.5%	74.1%	69.5%	0.18
Secondary^a^	26.4%	18.5%	25.9%	30.5%	
Ovarian reserve test					
AFC^b^	19.6±11.4	19.9±9.8	21.1±11.8	18.2±11.6	0.38
Basal FSH (mIU/ml)^b^	7.1±3.1	7.1±2.1	6.5±1.7	7.6±4.2	0.18
AMH (ng/ml)^b,c^	3.2±2.6	4.3±3.3	3.2 ±2.5	2.8 ± 2.1	0.03

No significant association was observed in terms of total gonadotropin requirement, duration of stimulation, oocyte yield, maturation rate, embryos fertilized, or number of grade-I embryos among the three groups (Table 3).

**Table 3:** Controlled ovarian hyperstimulation (COH) cycle characteristics ^a^Percentage ^b^Mean±SD FORT=Number of follicles ≥14 mm on the day of trigger/antral follicle count x 100 FOI = Number of oocytes retrieved/antral follicle count × 100 Maturation rate = Number of M2 oocytes/number of oocytes retrieved × 100 Fertilization rate = Number of embryos with two pronuclei/number of oocytes injected × 100

COH Cycle Characteristics	Whole Cohort (n=140)	NN (n=27)	NS (n=54)	SS (n=59)	P-value
Days of stimulation^b^	9.8±1.7	10.1±1.7	9.7±1.6	9.8±1.9	0.53
Total gonadotropin dose^b^	3348.1±1356.3	3255.6±1247.9	3343.2±1421.8	3394.9±1362.8	0.91
Total human menopausal gonadotropin (HMG) dosage ^b^	1136.8±930.9	1283.3±987.0	1047.2±865.73	1151.7±968.2	0.557
Oocytes retrieved^b^	14.1±9.6	15.6 ±8.7	15.3 ±10.7	12.5±8.7	0.209
Mature oocytes^b^	10.1±7.7	10.3 ±7.2	11.4 ±9.1	8.7 ±6.4	0.166
Maturation rate^a^	69.9%	65.6%	74.2%	67.9%	0.100
Oocytes injected^b^	9.7±6.2	9.8 ±5.0	10.6 ±7.2	8.8 ±5.8	0.32
Follicular output rate (FORT)^a^	62.0%	67%	59.4%	62.1%	0.61
Follicular output index (FOI)^a^	79.8%	83%	77.5%	80.2%	0.862
Embryos fertilized^b^	7.4±5.18	7.4±4.0	7.7±5.7	6.53±5.1	0.48
Fertilization rate (%)^a^	75%	78%	70%	77%	0.54
Grade-I embryos^b^	4.9±4.4	4.9±3.4	5.2±4.8	4.7 ±4.4	0.81

Of the 140 women, 128 women had undergone embryo transfer and were followed up for a maximum of two embryo transfer cycles. The cycle outcomes were comparable across the three groups (Table 4).

**Table 4:** Cycle outcomes ^a^Percentage ^b^Mean±SD

Embryo Transfer Parameters	Whole Cohort (128)	NN (n=27)	NS (n=54)	SS (n=59)	P-value
Total number of embryo transfers	166	30	58	78	-
Fresh embryo transfers^a^	15.1%	6.7%	13.8%	19.2%	0.25
Frozen embryo transfers^a^	84.9%	93.3%	86.2%	80.8%	
Endometrial thickness^b^	9.4±1.5	9.5±1.6	9.5±1.5	9.3±1.8	0.60
Number of embryos transferred^b^	2.7±0.71	2.8±0.83	2.68±0.79	2.58±0.73	0.36

The cumulative pregnancy rate, cumulative clinical pregnancy rate, and cumulative live birth rate were significantly higher in the NN group when compared to the NS and SS groups. The cumulative miscarriage rate and the ongoing pregnancy rates were comparable across the three groups (Table 5).

**Table 5:** Reproductive outcomes ^a^Percentage ^c^p<0.05

Reproductive Outcomes	NN (n=27)	NS (n=54)	SS (n=59)	P-value
Cumulative pregnancy rate^a,c^	82.7%	50%	38.2%	0.0005
Cumulative clinical pregnancy rate^a,c^	78.8%	44.7%	34.5%	0.0005
Cumulative miscarriage rate^a^	15.4%	18.09%	10.0%	0.44
Cumulative live birth rate^a,c^	50.0%	20.2%	20.0%	0.005
Ongoing pregnancy rate^a^	17.3%	14.9%	8.2%	0.39

## Discussion

This prospective cohort study was conducted to determine the genotypic distribution of LHCGR N312S SNP in infertile Indian women undergoing ART and to evaluate the impact of this polymorphism on ART outcomes.

In our study, 19.3% were homozygous for N312, 42.1% were homozygous for S312, and 38.6% were heterozygous for N312S. In contrast to our study, the percentage of women heterozygous for N312S was higher than homozygous for S312 in studies by Pirtea et al. [15] (48.1% vs. 29.2%), Ramaraju et al. [16] (45.6% vs. 34.7%), and Lindgren et al. (47% vs. 35%) [17]. This may be related to the smaller sample size of our study.

The baseline characteristics among the three groups were comparable. There was no significant difference in the indication of ART among the three groups. The percentage of women with PCOS was higher in the NN (26.0%) and NS (22.2%) groups compared to the SS group (13.5%). These findings matched those of studies by Singh et al. [12], Capalbo et al. [13], and Makhdoomi et al. [14], who found that the NN and NS groups were more susceptible to developing PCOS than the SS group. Thathapudi et al. [18] discovered that the SS group was more predisposed to PCOS, which contradicted our findings. The disparity across the studies might be because of ethnic or racial differences and the varied diagnostic criteria utilized for PCOS. Rotterdam’s criteria were used to diagnose PCOS in our study.

The ovarian reserve tests such as basal FSH and AFC were comparable among the three groups, but the AMH levels in the SS group (2.8±2.1) were significantly lower than in the NN (4.3±3.3) and NS groups (3.2±2.5) (p=0.034). This may be attributed to the percentage of women with decreased ovarian reserve (AMH < 1.1 ng/ml or AFC < 5) being higher in the SS group (27.1%, 16/59) than in the NS (24.1%, 13/54) and NN (7.4%, 2/27) groups. Lindgren et al. [17] found no significant difference in baseline FSH and AFC across the three groups as we did. However, they did not compare AMH levels.

There was no significant difference in COH cycle features such as total gonadotropin dosage, duration of COH, oocyte yield, fertilization rate, or number of grade I embryos, which supports the results of the study by Pirtea et al. [15]. Unlike the aforementioned findings, Ramaraju et al. [16] found that the women in the NS and SS groups had statistically higher requirements of r-hLH (p=<0.0001) compared to women in the NN group and the total number of oocytes retrieved, mature oocytes, and good-quality embryos was significantly higher in the NN group compared to the other two groups. This discrepancy can be because of differences in the IVF protocol used, the type of LH supplementation (HMG or r-hLH), and the timing and dosage of LH used. In our study, we started HMG from day 1 of COH along with FSH for patients aged ≥35 years, with decreased ovarian reserves, or with previous history of IVF failure.

When subgroup analysis was done, in 24 (17.14%) women, we had to increase the gonadotropin dose mid-cycle because of hypo-responses to stimulation, of which 45.84% were in the SS group (n=11), 33.3% were in the NS group (n=8), and 20.83% were in the NN group (n=5), but it was not statistically significant (p=0.845).

When we compared reproductive outcomes, the cumulative pregnancy rate (82.9% vs 50.0% vs 38.2%, p=0.0005), cumulative clinical pregnancy rate (78.8% vs 44.7% vs 34.5%, p=0.0005), and cumulative live birth rate (50.0% vs 20.2% vs 20.0%, p=0.005) were statistically higher in the NN group than the NS and SS groups, respectively. Like our study, Javadi-Arjmand et al. [19] carried out a case-control study of the Iranian infertile population undergoing IVF and found that the NN group had a protective effect against IVF failure (p=0.03 and OR=0.04), while the SS group was susceptible to IVF failure (p=0.003 and OR=3.88).

Lindgren et al. [17] conducted a cross-sectional study in which women with S312 in LHCGR had significantly higher pregnancy rates (OR=1.61, 95%, p=0.008) after IVF than those in N312 or N312S, unlike our study, and they had not followed the patients for live birth. This variation can be because of racial differences and different IVF protocols like agonist and antagonist were followed in their study. We used only the GnRH antagonist protocol in our study. Pirtea et al. [15], in whose study ART with PGT-A was done, found that LHCGR N312S SNP was not predictive for clinical or live birth outcomes, which is in contrast to our study. Only 15.4% were Asians in the aforementioned study, and PGT-A was not performed.

Ramaraju et al.’s [16] study demonstrated that the clinical pregnancy rate after excluding patients with PCOS and endometriosis was significantly higher in the SS and NS groups compared to the NN group (56% vs 57.1% vs 40.8%, p=0.04), but there was no difference in live birth rates among the three groups. The long GnRH agonist protocol was followed in this study.

In our study, a subgroup analysis was conducted excluding women with decreased ovarian reserves (n=102), and comparable outcomes were noted in terms of baseline characteristics, ovarian reserve tests, and COH cycle outcomes. In terms of reproductive outcomes, women in the NN group had significantly higher cumulative pregnancy rates (81.25% vs 59% vs 42.5%, p=0.004), cumulative clinical pregnancy rates (77.1% vs 51.3% vs 37.5%, p=0.004), and cumulative live birth rates (50% vs 25% vs 23.75%, p=0.04) than those in the NS and SS groups, respectively. 

The strengths of our study are its prospective design, inclusion of women of only Indian ethnicity, adoption of GnRH antagonist protocol for all patients, and at least one good embryo was transferred in all embryo transfer cycles. The limitations of the study are the small sample size and investigation of only one SNP: LHCGR N312S(rs2293275).

Studies with a large sample size of Indian infertile women undergoing ART with GnRH antagonist protocol are required to confirm the findings of our study and to assess the impact of LHCGR N312S SNP on ART outcomes. We need more studies on individualized/personalized COH using pharmacogenomics for FSH and LH supplementation based on combined FSH and LH receptor SNP and to assess their effects on ART outcomes.

## Conclusions

To conclude, even though demographic characteristics and COH cycle outcomes were comparable among the three groups, women who were homozygous for N codon had significantly higher cumulative pregnancy rates, cumulative clinical pregnancy rates, and cumulative live birth rates than women who were heterozygous for N312S and homozygous for S codon, even after excluding women with decreased ovarian reserves. Hence, assessing LHCGR N312S SNP may help us to predict reproductive outcomes in ART and aid in better counseling for infertile couples.
